# A soft, synergy-based robotic glove for grasping assistance

**DOI:** 10.1017/wtc.2021.3

**Published:** 2021-04-20

**Authors:** Ryan Alicea, Michele Xiloyannis, Domenico Chiaradia, Michele Barsotti, Antonio Frisoli, Lorenzo Masia

**Affiliations:** 1 Assistive Robotics and Interactive ExoSuits (ARIES) Lab, Institute for Computer Engineering (ZITI), Heidelberg University, Heidelberg, Germany; 2 Sensory-Motor Systems (SMS) Lab, Institute of Robotics and Intelligent Systems (IRIS), ETH Zurich, Zurich, Switzerland; 3 The Spinal Cord Injury Center, University Hospital Balgrist, University of Zurich, Zurich, Switzerland; 4 Perceptual Robotics (PERCRO) Laboratory, TeCIP Institute, Scuola Superiore Sant’Anna, Pisa, Italy

**Keywords:** Wearable robotics, assistive technology, exosuit

## Abstract

This paper presents a soft, tendon-driven, robotic glove designed to augment grasp capability and provide rehabilitation assistance for postspinal cord injury patients. The basis of the design is an underactuation approach utilizing postural synergies of the hand to support a large variety of grasps with a single actuator. The glove is lightweight, easy to don, and generates sufficient hand closing force to assist with activities of daily living. Device efficiency was examined through a characterization of the power transmission elements, and output force production was observed to be linear in both cylindrical and pinch grasp configurations. We further show that, as a result of the synergy-inspired actuation strategy, the glove only slightly alters the distribution of forces across the fingers, compared to a natural, unassisted grasping pattern. Finally, a preliminary case study was conducted using a participant suffering from an incomplete spinal cord injury (C7). It was found that through the use of the glove, the participant was able to achieve a 50% performance improvement (from four to six blocks) in a standard Box and Block test.

## Background

One of the great philosophers of antiquity, Aristotle, once named the hand “the tool of tools,” and in truth, the human hand is unmatched in its synthesis of dexterity, strength, sensitivity, and versatility. Proper functioning of the hands is such a critical element of the human experience, that many able-bodied persons find it difficult to imagine what life would be without their complete use.

Unfortunately, many people experience this scenario firsthand when dealing with neuromuscular impairments as a result of a spinal cord injury (SCI). In the United States alone, there are an estimated 17,810 new SCIs occurring annually, with more than 50% of these injuries impairing upper body function (Wyndaele and Wyndaele, 2006; Jain et al., 2015). These sorts of injuries are especially detrimental, as they can critically harm a person’s independence by affecting their ability to perform activities of daily living (ADLs) (Snoek et al., 2004). Even minor hand impairments can severely affect one’s ability to carry out useful employment by impacting grip strength and overall manual dexterity (O’Sullivan and Colville, 1993).

In the case of SCI, postrehabilitation employment has long been considered a critical metric by which to judge the success of SCI rehabilitation (Guttmann, 1954; DeVivo et al., 1987; Ottomanelli and Lind, 2009). Unfortunately, even up to 10 years after discharge from rehabilitation programs, between 60 and 80% of SCI patients remain totally reliant on external assistance programs due to lack of employment (DeVivo et al., 1987; Levi, 1996; Lidal et al., 2007; Ottomanelli and Lind, 2009; Young and Murphy, 2009). The gravity of this finding is only fully understood when considering that poor vocational outcomes are strongly correlated with the onset of postinjury depression and/or substance abuse, along with reduced patient longevity (Trieschmann, 1980; Yerxa and Baum, 1986; Krause, 1990; Tate et al., 2004).

It is therefore critically important for this population to have access to new rehabilitation techniques that will better prepare them for re-entry into the workforce, and ultimately, a higher quality of life.

With this goal at the forefront, a variety of robotic devices have been proposed within the last decade to assist in the rehabilitation of the SCI population. Many of these proposed devices were constructed of stiff and rigid materials such that high levels of force assistance and wide control bandwidths could be achieved (Ryu et al., 2008; Arata et al., 2013; Jones et al., 2014). Although many of these systems are capable of producing adequate forces required for rehabilitation assistance, the rigid nature of their construction introduces many impracticalities that are incompatible with use in a patient’s daily life. For example, misalignment of the device with the joints of its wearer will negatively impact the comfort of the patient and performance of the device (Perry et al., 2007; Nef et al., 2009). Additionally, the rigidity of the structure introduces additional mass, inertia, and volume to the system. This presents particular challenges when designing devices for the hand which is very sensitive to any excess mass or bulk (Aubin et al., 2013).

Another consideration is that while rigid assistive systems are frequently developed for use in a clinical setting (Cerasa et al., 2018; Serrano et al., 2018), a large percentage of patients will never fully recover their motor ability after an SCI (Fawcett et al., 2007). Systems which are too complex or bulky to be used outside of a clinician’s office will be of little use to this class of patient.

These challenges have sparked an increasing interest in developing soft, wearable robots made of fabric (Awad et al., 2017; Yap et al., 2017; Gerez et al., 2019) or elastic polymers (Polygerinos et al., 2015; Kang et al., 2019), to transmit forces to the human body. The intrinsic compliance and adaptability of these soft systems offers many ergonomic advantages and may overcome some of the limitations present in rigid rehabilitation robotic systems. For instance, their soft nature allows them to fit closely to the body and removes much of the challenge associated with joint alignment. Additionally, these flexible systems are lightweight by nature, and require little mass and bulk to be placed on the actuated joints themselves, making them ideal designs for providing assistance to distal regions of the body, such as the hand. An additional hope is that the reduced size and weight of these devices will also allow for more portability, and thus potentially expand rehabilitation use in the home of these patients supplementary to that in the clinic.

These soft, wearable systems have been successful at applying meaningful assistance to a variety of joints (Asbeck et al., 2015; Lessard et al., 2018; Lotti et al., 2020), as well as to the hand itself (Polygerinos et al., 2015; Dwivedi et al., 2019; Kang et al., 2019; Park et al., 2020). When considering applying forces to the hand, a popular approach has been to support as many degrees of freedom (DoFs) as manageable such that a wide subsection of grasps and postures can be supported (Cappello et al., 2018; Hameed et al., 2018; Rose and O’Malley, 2018; Fajardo et al., 2019). This approach is only achievable by using additional actuators, which can quickly add an unacceptable level of complexity to the mechanical system worn on the hand. Additionally, any increase in the number of actuators results in a corresponding increase in both power requirements, dimensions, and mass of the complete system. Balancing between actuation complexity and system mass is a critical consideration in the development of wearable systems.

To design for and understand the mechanical complexity of the human hand, with more than 24 DoFs, grasp synergies have become a fundamental solution toward reducing this high dimensionality (Liu et al., 2016; Gracia-Ibáñez et al., 2020). The wide variety of hand postures can be deconstructed into a small number of principal components, the first two of which account for 84% of the variance of ADL grasp postures of common objects (Santello et al., 1998). In an effort to draw a middle ground between system complexity and user functionality, it is posited here that by designing an assistive device around the concept of postural grasp synergies, the number of actuators in the system may be reduced while retaining the ability to provide support across a broad subset of ADLs. The actuation complexity of such a device could be further reduced by utilizing passive elements to provide assistance for specific DoFs.

**Figure 1.**
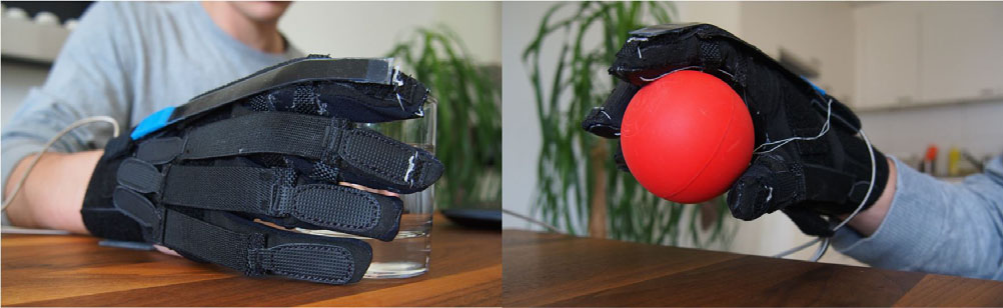
The soft, synergy-based robotic glove for grasping assistance.

The solution proposed here offers an initial step toward realizing an impactful degree of manual dexterity with a mechanically simple system by integrating postural synergies into the design. To demonstrate the practical viability of such a system, the design and characterization of a soft, underactuated glove built around this principle of postural synergy is presented toward the assistance of finger flexion/extension and grasp support (Figure 1). Such a system presents a greatly reduced complexity in both design and control while maintaining the capability to provide assistance for a diverse array of everyday motions. Actuation and power transmission efficiency within this system are characterized, and force output capability is examined. Finally, we assess the effectiveness of the device in a single case study with a patient suffering from an incomplete SCI.

## Design

### Design Objectives

The targeted rehabilitation group of this design is patients who are suffering from partial SCI with low to moderate levels of muscle spasticity (Modified Ashworth Scale 0–2). The design objectives of this device (Table 1) were determined by examining the baseline dynamic and kinematic requirements required to perform many necessary ADLs (Hume et al., 1990; Weir, 2002; Pylatiuk et al., 2006).

**Table 1.** Design requirements

Design characteristic	Glove	
	Fingers	Thumb
Range of motion	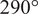	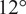
Fingertip force	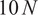	
Bandwidth	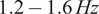	
Distal mass	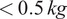	

When creating devices to be worn on the hand, it is critically important to maintain simplicity of design and to utilize efficient space management in order to avoid unnecessary weight and complexity. This is especially difficult because of the high number of DoFs that are associated with dexterous hand movement. Although counterintuitively, a large fraction of ADLs do not require fine control of each DoF of the hand. There is increasing evidence that many movements used in the large variety of ADLs involving the hand can be deconstructed into a narrow set of coordinated postural synergies (Santello et al., 1998; Della Santina et al., 2017; Gracia-Ibáñez et al., 2020).

### Glove Design

The device was designed specifically to target patients suffering from mild to moderate SCI. Specifically, the target population of the device is these patients who retain some degree of residual limb function. With this in mind, the glove was fabricated with materials specially chosen for user comfort as well as efficiency of power transmission. Special considerations, such as an open palm section, were made to assist the ease of donning and doffing of the glove by a motor-compromised patient.

In order to reduce the mechanical complexity of the system, the current design provides active assistance for finger flexion only. Only three digits are actively supported, these being the thumb, index, and middle fingers. The support of the thumb is required, given its unique anatomy and fundamental role in all grasps that require opposition. The index finger is fundamental for precision manipulation (Cutkosky, 1989), and the middle finger is responsible for the second highest force production generated during prehensile power grasps (Santello and Soechting, 2000). While the ring and little fingers have a secondary role in stabilizing the grasp of big objects, they are not as critical to grasp capability. The importance of these three fingers is also clear from the principal component analysis conducted in the works of Santello (Santello et al., 1998) and Ingram (Ingram et al., 2008) in which the eigenvalues of the thumb, index, and middle finger joints on the sagittal plane are the major contributors to the first two principal components.

**Figure 2.**
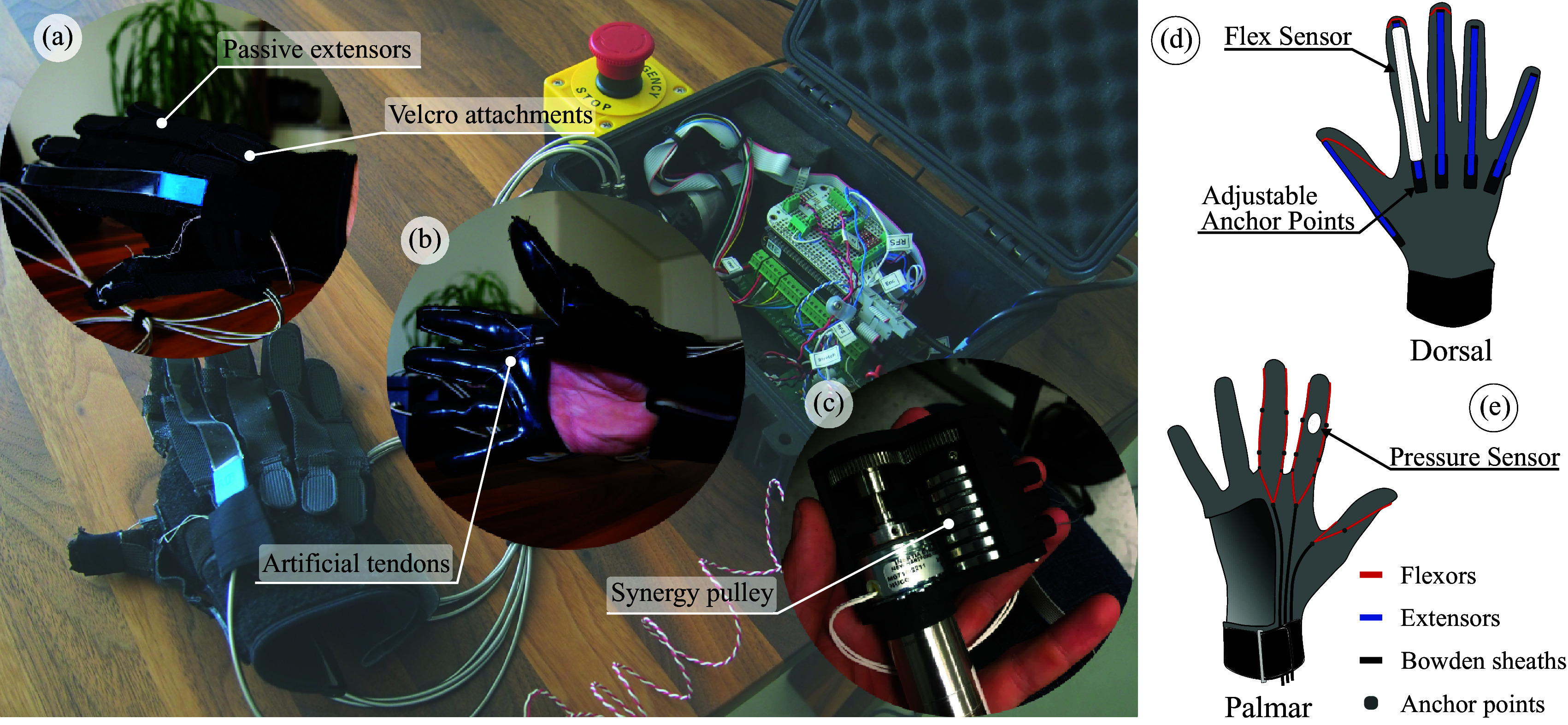
(a,b) Photos of the worn glove from dorsal and palmar viewpoints. (c) Photo of the postural synergy-inducing spool. (d,e) Illustration of the synergy-based, robotic glove. Diagram detailing the transmission paths and sensor placement of the synergy-based glove system. Coiled Bowden sheaths (black) are rigidly anchored on the palmar side of the hand. The tendons (red) extend from these points and are routed on the sagittal plane of each respective finger before rigidly anchoring at its tip. Anchor points (dots) restrain the tendons to the glove along these routing paths and allow for torques to be generated about each joint of the finger. Passive extensor bands (blue) coerce the fingers into an extended position while relaxed. The anchor points of these extensors are attached by Velcro and may be easily adjusted with the off-hand to fit the preferences of the wearer.

Finger extension assistance is provided through passive spring elements fixed onto the dorsal tip of each finger (Figure 2), and tension in these elastic elements encourages the hand into a naturally open position when relaxed, eliminating the need for an antagonistic actuation of the fingers’ extension. The anchor point of these tensioners may be comfortably adjusted using hook and loop (Velcro) straps, with only one hand; in this way, a patient with residual motor capacity in their contralateral hand may have personal and direct control over the amount of finger extension assistance received and the level of comfort during use. Because muscle spasticity is a common ailment of SCI patients, these passive elements allow for the added opportunity to passively stretch spastic finger flexors, a common rehabilitation method for spasticity relief (Smania et al., 2010).

A wrist strap sewn on to the base of the glove (Figure 2) provides additional comfort and security for the wearer during use. It accomplishes this by anchoring the glove to the wrist and reducing shear forces by loading the carpal bones normally and maintaining alignment of the Bowden cables with the direction of the wrist. The size of the glove can only be adjusted by loosening and tightening the strap. Other size adjustments are not possible. The three independent Bowden cables terminate above the wrist inside the palm of the glove. The internal tendons are routed along the palmar side of the fingers before finally anchoring to the dorsal tips of the thumb, index, and middle fingers.

### Actuator Design and Power Transmission

When considering any device to be worn on the hand, mass is a critical consideration. Previous studies have determined that added mass to the hand should not exceed approximately 450 g, after which, adverse effects may be observed (Aubin et al., 2013; Gasser and Goldfarb, 2015). Of course, this limit is highly subject-specific and care was taken to keep the mass of glove device well under this value. Thus, the system presented in this work only adds 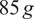 of mass to the hand and wrist. In an effort to keep the added mass low, torques are delivered to the joints of the wearer through the use of a tendon-driven system such that actuators and bulky transmission equipment can be located away from the hand. Tendons of braided, high-test, Dyneema wire (0.5-mm 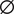 ) are rigidly fixed to the dorsal tips of each actuated finger, such that wire tension induces a torque across each biological joint (Xiloyannnis et al., 2016). These tendons are routed through low-friction PTFE tubing (1.5-mm ID) which is shielded by helical Bowden sheaths (4-mm OD, *Asahi*, Tokyo, Japan). After exiting the Bowden sheaths, additional PTFE tubing and PTFE-embedded fabrics were used at all locations where wire–glove contact was expected to occur, thereby reducing friction wherever possible.

With a fixed anchoring at the dorsal tip of each finger, the three tendons are kinematically linked to the hand, actuating the thumb, index, and middle fingers. All tendons are controlled by a single actuator (Maxon Motors EC-i 40); thus, the system is underactuated. An intrinsic feature of a tendon-driven system is that it imposes very soft constraints on the wearer while unpowered. This allows an impaired wearer the freedom to choose to utilize from using other grasp types, such as the commonly observed lateral pinch type grasp (García Álvarez et al., 2017), if the situation calls for it.

The actuation unit, which weighed less than 1 kg, was designed as a desktop functional unit. For wheelchair users, we do not anticipate to provide a body-worn solution, as the extra load could be borne by the wheelchair’s frame. In the future, a wearable solution may be designed with the actuation unit in a proximal position, that is, carried in a belt or as a backpack.

#### Soft postural synergy

The human hand has 15 joints and 20 DoFs. This high dimensionality is partly responsible for its unparalleled dexterity but raises unsolved control and design challenges. This is why, when emulating or assisting the human hand, a practical and convenient approach is that of using fewer actuators than DoFs.

There are various ways to do so, including rigid or compliant couplings between DoFs or differential mechanisms (In and Cho, 2015). A more principled approach, and one that has proved its effectiveness for robotic and prosthetic hands (Catalano et al., 2014), is that of embedding hand postural synergies in the mechanical design of the device. The idea of postural synergies arose from the discovery that a remarkably small number of kinematic coordination patterns of the hand explain a high fraction of the variance in everyday hand movements—60% for the first synergy alone (Santello et al., 1998). By leveraging this concept, the use of a small number of key motion patterns may be capable of supporting a wide variety of ADL grasp postures. While this idea is fascinating, the mechanical implementation of postural synergies requires rigid couplings between DoFs of the hand, thus limiting the adaptability of the hardware to a wider variety of movements.

**Figure 3.**
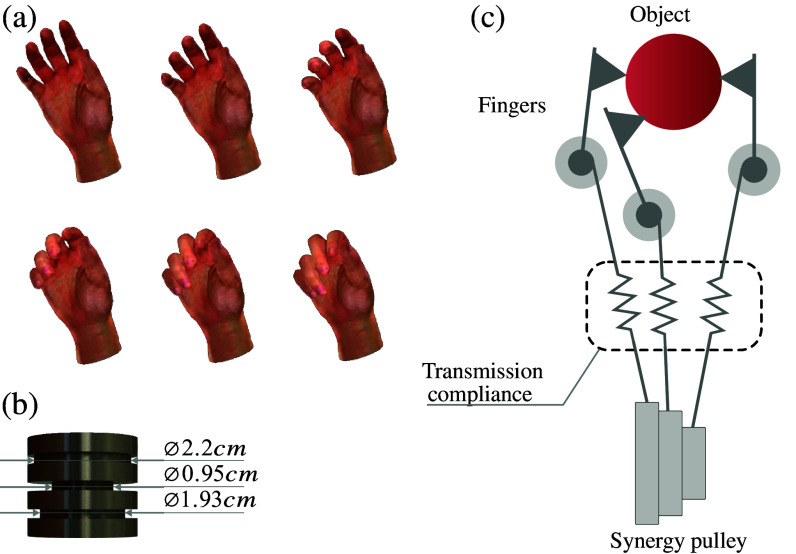
(a) Renderings of the progression of the first postural synergy of the hand. The top-left frame depicts the hand in the relaxed state, whereas the bottom-right frame depicts the hand after it has moved through the maximum range of the first postural synergy in the positive direction (flexion). Images of the hand were rendered using the LibHand library (Šaric, 2011) and the dataset recorded by Santello et al. (1998). (b) The multichannel pulley, which supported the synergistic motion patterns, has different diameter channels for the thumb, middle, and index fingers. (c) The principle of soft postural synergies proposed in Bicchi et al. (2011), as applied to the soft robotic glove. The hard constraints imposed by the design of the pulley are softened by the compliance of the fabric and cable transmission; this increases adaptability of the glove to grasping objects of different shapes.

Bicchi et al. (Bicchi et al., 2011; Catalano et al., 2014), however, showed that combining postural synergies with compliance in the transmissions leads to an elegant solution to this problem: if chosen appropriately, the compliance allows for adaptability and versatility in grasping with an extremely simple design. The concept of soft synergies described in Bicchi et al. (2011) is shown in Figure 3c.

In the system presented here, the first postural synergy of the human hand was embedded into the design of the actuation module; compliance in the transmission (Bowden cables and fabric) softens this constraint. The mechanical implementation of the postural synergy consists of a multichannel pulley, designed to anchor each of the three tendons to the motor. The channels were designed with a ratio between diameters, extracted using the method described in Xiloyannnis et al. (2016), so as to move the thumb, index, and middle fingers according to the first hand synergy. The geometry of the pulley is shown in Figure 3b.

**Figure 4.**
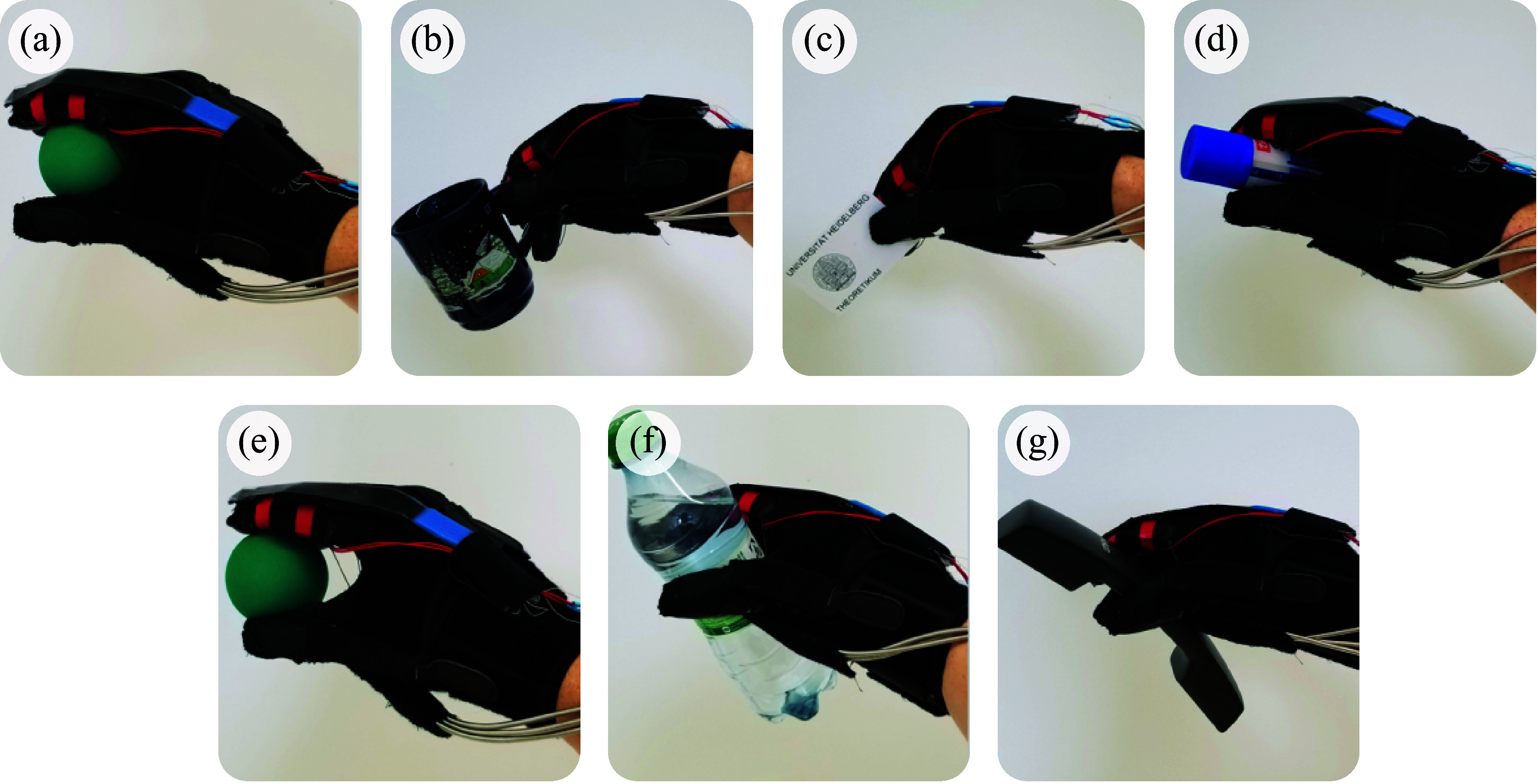
(a–g) Example of graspable objects using the underactuated, synergy-based glove system. In spite of the single DoF, the synergistic nature of glove actuation allows for motor-compromised patients to maintain a secure grasp over a wide variety of common items.

This coordination pattern is the one movement that, by definition, explains most of the variance in daily grasping activities. That is, the one underactuation strategy that would constrain the hand the least during routine use, allowing for the exertion of cylindrical, lumbrical, and pinch grasps (Figure 4).

## Device Performance

The performance of the device is highly dependent on both the efficiency of its design and its positioning on the patient’s body (Quinlivan et al., 2015). Because of this, it is beneficial to understand how the suit will behave in a realistic use scenario. Due to the underactuated nature of the device, its soft structure, and the underlying compliance of the human tissue, it is not possible to derive a closed-form, analytic solution relating force at one point in the glove to the current delivered to the motor. For this reason, a characterization of the wearable system was performed in order to relate the overall grasp force to motor current for two types of grasps, the pinch grasp (fine and small forces) and the cylindrical grasp (higher forces). Additionally, the individual stiffness of the transmission elements, the internal friction of the transmission under various loading conditions, and the internal backlash of the transmission were all characterized experimentally prior to testing with the patient.

### Transmission Stiffness

The transmission system is made of two primary components: the outer Bowden sheath, and the inner, actuated tendon. Both of these components exhibit deflection while loading the exosuit; therefore, it is necessary to characterize this aspect, so that these control disturbances and nonlinear behaviors can be better understood. Deflection of the tendon element manifests throughout loading as extension: in the case of the Bowden coil, this deflection manifests as compression.

**Figure 5.**
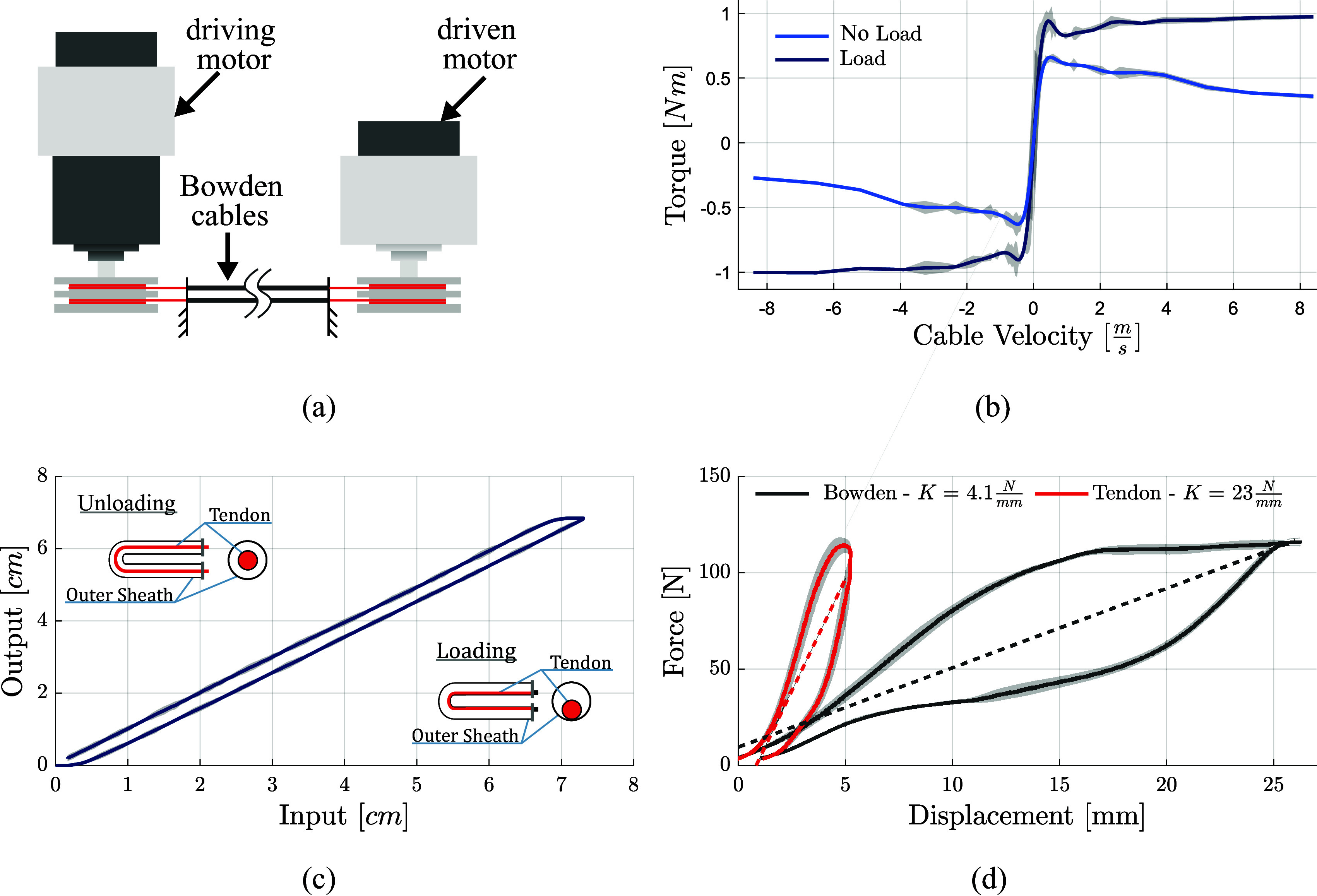
(a) Illustration of the test bench used for characterizing the Bowden cables. (b) Friction of the transmission system was characterized across a range of velocities. Each curve represents the different loading conditions of the motor as it executed its velocity trajectory. The blue curve (inner) represents the friction of the internal motor dynamics only. The black curve (outer) demonstrates the additional friction imposed by a load of 50 N. (c) Backlash characterization of the Bowden cable transmission system with a 180°-bend angle. Desired position is depicted against the input position for 10 cycles. Subfigures provide visualizations of the root mechanism of backlash within the system using longitudinal (left) and transverse (right) cross sections. (d) Stiffness characterization of primary transmission elements. Stiffness estimations were made by applying a singular loading profile to the system over 10 repetitions. Dashed lines indicate a best-fit line for each transmission element, the slope of which is the approximate equivalent stiffness of the transmission element. These values are used as stiffness approximations when modeling the system and are annotated in the legend. Note that the displacement of the Bowden coil (black) was measured in compression, whereas the displacement of the tendon (red) was measured in extension. (*All shaded regions encompass one standard deviation of recorded values.*)

One end of each component was rigidly fixed to a testing block. For the purposes of evaluating the component stiffnesses, during these experiments, the free end of the tendon was secured to steel cable, assumed inextensible, which was spooled onto a direct-drive actuator. Additionally, this analysis was also performed for the elastic elements which were fixed to the dorsal side of each finger. The experimental arrangement for both cases is shown in Figure 5a.

For each test, the motor was sent a trajectory of position commands. The forces exerted on the component being measured were estimated using the holding current passing through the motor. In the case of the tendon trials, tensile forces were applied directly to the component in the absence of the Bowden sheath such that the elongation of the tendon unit was measured through motor position. The Bowden sheath trials were done under the assumption that the tendon is significantly stiffer than the Bowden sheath. While within the sheath, a tensile load applied to the tendon induces compressive displacements within the Bowden sheath which were then measured using the rotation of the motor. The Bowden sheath was supported such that it remained horizontal throughout the tests.

Passive parasitic forces of the spring elements within the suit were identified independently for both the tendon itself and the Bowden coil. Both elements demonstrated nonlinear characteristics and hysteresis during loading conditions, although the magnitude of hysteresis of the Bowden coil in compression exceeded that of the tendon in tension. Figure 5d depicts these results. Although the tendon demonstrated a high spring constant relative to the coil, it deformed considerably under load. This, when considered with the hysteresis bands which arose during unloading, indicates that these tendons cannot be modeled as inextensible, nor can they be considered to be purely elastic in practice. Although it is noted that the linear regions of the tendon stress–strain relationship have very nearly the same slope. This indicates that the stiffness of these tendons is much more predictable while subjected to variable load conditions.

The stress–strain relationship of the coil is much more nonlinear, demonstrating a much greater hysteresis band, and thus, a much more substantial loss of energy. In this case, loading and unloading results in large differences in stiffness constant, and correspondingly energy losses.

**Figure 6.**
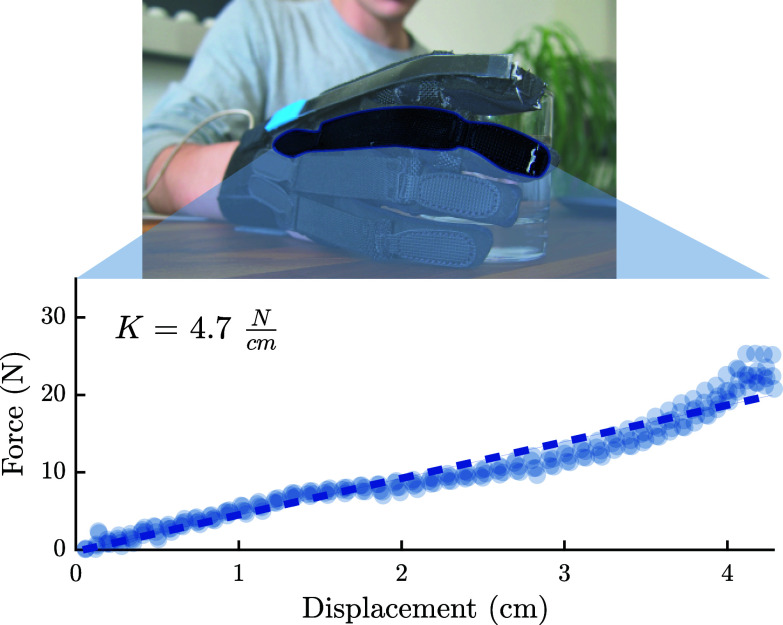
Measured stiffness of the adjustable, elastic elements.

The characterization of the passive elastic elements, shown in Figure 6, demonstrates an approximately linear behavior. Contrarily, the large degree of hysteresis observed in the transmission elements is a potential source of inefficiency of the transmission system (Figure 5d).

### Transmission Friction

An equally important consideration is the effect of any internal friction within the transmission system during normal wear conditions. The tendon–Bowden sheath interaction is a primary source of friction in the exosuit system (Jeong and Cho, 2015; Hofmann et al., 2018). For this reason, it is important to understand the effects of this interaction.

To do this, current through the motor was used to estimate the torque being delivered as the motor executed a trajectory of command velocities. This trajectory was executed under different conditions: no-load and no sheath, Bowden sheath only, and Bowden sheath with a constant 50-N load. The no-load, no sheath condition was examined to quantify the friction within the gearmotor system. To evaluate the latter two conditions, a testing bench was assembled in which a tendon–Bowden system was engaged with the motor spool unit. In the case analyzing the internal friction with an additional 50-N load applied, the complete Bowden system terminated at a secondary motor-pulley system, which was used to simulate a constant 50-N load on the tendon.

A major source of inefficiency within the system was expected to arise from internal friction within the Bowden system. As such, this was examined as a part of this study as well, although in a preliminary fashion. As expected, in general use conditions, results indicate that internal friction of the transmission system is nontrivial. Measured internal system friction increased under increased loading conditions of the exosuit. Results are shown in Figure 5b.

### Transmission Backlash

The degree of backlash hysteresis inherent to Bowden transmission systems was also investigated and is referred to simply as backlash in this context (Do et al., 2015). Because of the nature of the transmission, some degree of backlash is expected in the system and may be compensated for through control (Dinh et al., 2017a). Considerations for backlash are especially important when using position-based control methods. To identify the amount of backlash present in the system, a testing rig was developed in which the Bowden sheath was routed through a 3D-printed structure designed to enforce two separate 90°-bending angles for a total wrap angle of 180°. Two motor–spool pairs, located at each end, were engaged with the same tendon running through the sheath. The driving motor, located at the input of the sheath, wound the tendon according to a trajectory of position commands. The driven motor, located at the opposite end, was used to measure output position of the tendon.

Transmission backlash within tendon-driven systems remains a major problem in soft, wearable exosuits. Analysis of the transmission backlash revealed the presence of a near constant position error of 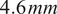 . This error was persistent throughout unloading. Clearly visible in Figure 5c, this backlash behavior is a consequence of the changing position of the tendon within the Bowden sheath, as the actuator changes directions from flexion to extension.

### Grasping Characterization

Finally, the holistic relationship between input current and force exerted at device output was identified through the use of a 3D-printed dynamometer. For these experimental system characterizations, an untrained, healthy subject was asked to wear the exosuit glove without resisting the movement of the device. This was done in order to assess the isolated behavior of the transmission system in realistic use conditions.

**Figure 7.**
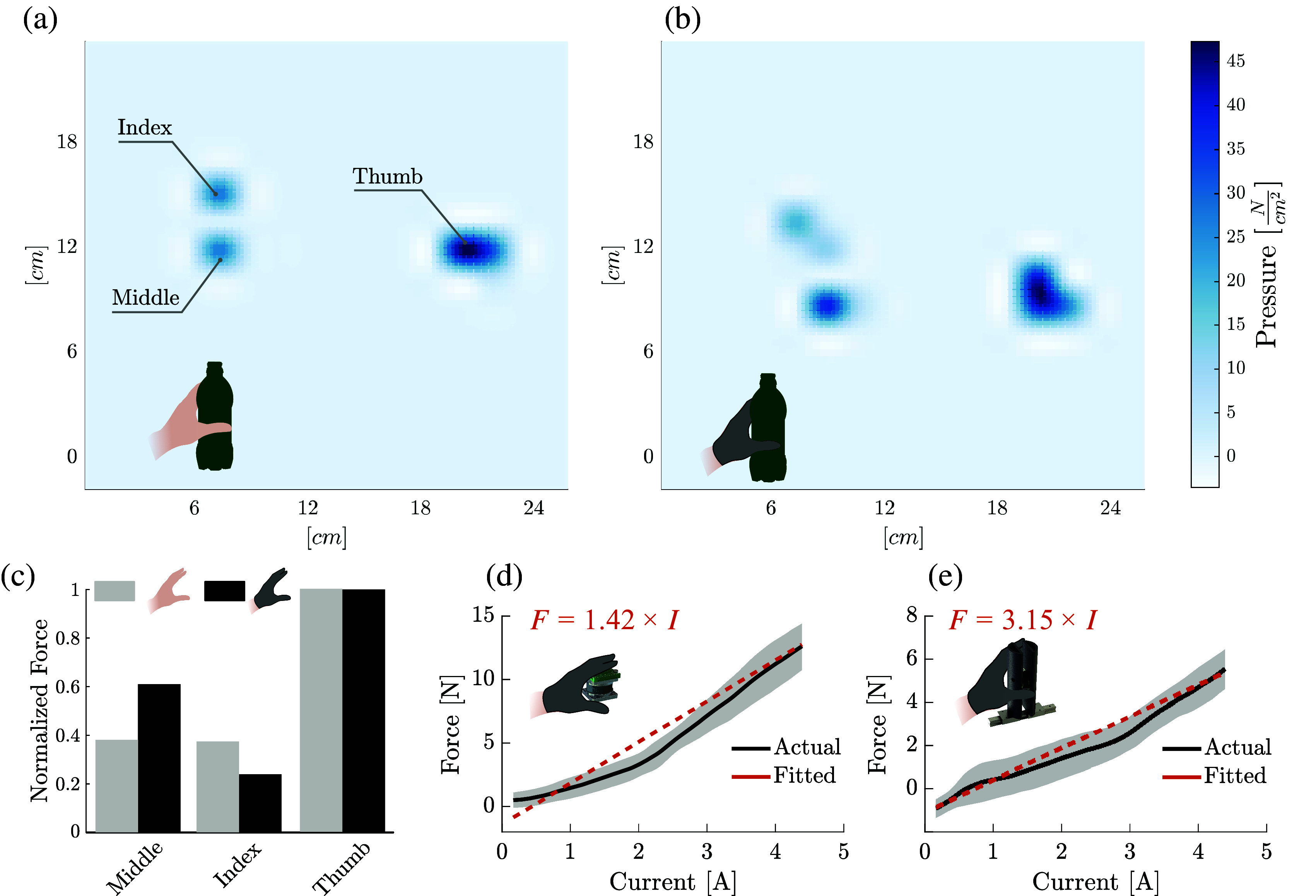
(a,b) Representative examples of pressure distributions measured using an instrumented cylinder while grasped by a healthy subject in the pinch configuration in the gloved and ungloved conditions, respectively. (c) Bar charts showing the normalized magnitudes of each pressure peak from (a,b) in more quantitative detail. (d,e) Plots of the force–current relationship of a relaxed, healthy subject in the gloved condition grasping a dynamometer for pinch and cylindrical grasps, respectively. Pressure distributions of a healthy subject’s grasp on the instrumented cylinder remained roughly the same both with and without the glove. The glove condition appears to shift a marginal fraction of the grasp pressure from the thumb to the other fingers, primarily the index finger. Output fingertip forces of the glove show a linear and repeatable relationship with motor current for both pinch and cylindrical grasp types. The slope of the force–current relationship appears to shift with grasp type.

During these trials, the device was actuated with a ramp input, as the participant wearing the device was instructed to place their palm around the dynamometer. The outer shell of the device was 3D-printed and housed an internal load cell (OMD-30-FE-450 N, *Optoforce*, Budapest, Hungary) for grasp force measurement. The subject was instructed to grip the device using both pinch and cylindrical grasp configurations, and to not resist or supplement the applied forces from the glove. In this way, 20 datasets were recorded for each type of grasp. Diagrams of the experimental setups and the dynamometer are depicted in Figure 7d,e.

Another important analysis is the manner in which the forces are distributed across the hand. In doing this, an untrained, healthy subject was asked to grasp a book-shaped object instrumented with an array of 8 × 8 force sensors (Small HSA, *Next Gen Ergonomics*, Pointe Claire, QC, Canada) using whatever grip strength felt comfortable. This was done both with and without glove assistance. In this way, any changes which the glove imparts on the distribution of grasp forces across the fingers could be examined.

The results of the output force characterization, which are shown in Figure 7d,e, indicate a nearly linear relationship between motor current and force output at the finger. Although it is important to consider that this linear relationship varies depending on the grasp type, the accuracy of these linear predictions have been evaluated using root mean square (RMS) error ( 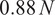 for pinch and 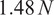 for cylinder) and 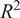 index ( 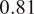 for pinch and 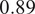 for cylinder). The measured forces delivered by the device reached up to 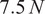 at finger level and up to 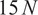 at palm level when the maximum current was provided to the motor.

Additionally, when worn by a healthy subject, it was found that the additional assistance provided by the glove had interfered little with the overall distribution of grasp pressure. These results indicate that while wearing the glove, the observed pressure distribution was roughly the same as grasps of the glove-free condition, although, in the glove case, there were slightly higher force contributions observed from the fingers than the thumb.

## Human Testing

**Figure 8.**
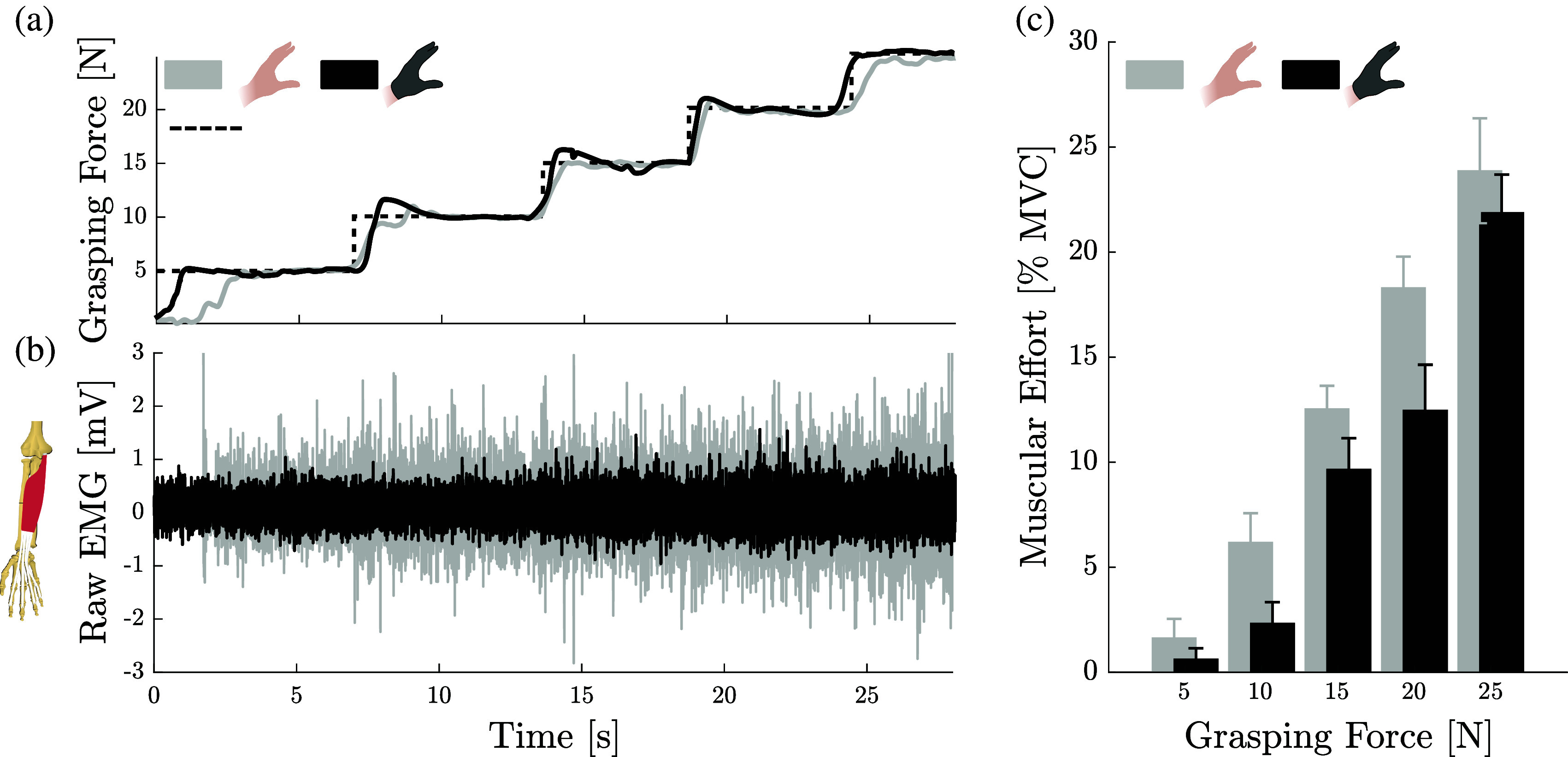
Effect of assistance from the glove on the activity of the flexor digitorum superficialis (FDS) muscle during a force-tracking task. Two healthy participants were instructed to grasp a dynamometer and following a reference grasping force trajectory, shown on a screen, with and without assistance from the glove. Assistance from the glove was controlled using a handheld joystick, whose position was proportional to the current of the motor. (a) Reference and measured force profiles for both conditions. The dashed line indicates the reference tracking trajectory. The solid lines indicate measured participant force profiles. (b) Examples raw muscular activity of the FDS during the force-tracking tasks from one participant. (c) Root mean square of the FDS activity during the tracking task, averaged over participants, for five different grasping forces, as a percentage of maximum voluntary contraction.

To quantify the effect of the active assistance from the glove while grasping an object, we monitored the electromyographic (EMG) activity (Trigno, Delsys, Natick, MA, USA) of the flexor digitorum superficialis (FDS) muscle, while gripping a sensorized object (OMD-30-FE-450 N, *Optoforce*). This was done with and without the glove at five equally spaced levels of grasping force between 5 and 25 N (Figure 8a). During the assisted condition, participants controlled the glove’s grasping force using a thumb-driven joystick, held in their left hand, whose position was proportional to the torque applied by the motor. The experiment was performed by two healthy participants. Placement of the electrodes and skin preparation was done in accordance to the SENIAM standards (Surface ElectroMyoGraphy for the Non-Invasive Assessment of Muscles) (Hermens et al., 2000).

Figure 8b presents the raw EMG activity of one of the two participants, in both conditions, showing a clear difference in the magnitude of the signal. Figure 8c reports the RMS of the EMG activity, for each force level, normalized by the participant’s maximum voluntary contraction and averaged across participants. Assistance in grasping results in an overall reduction of 22.8% of the activity of the FDS, with a decreasing benefit for forces above 15 N, where the device’s torque production capabilities saturate. These findings confirm that the device can work in parallel with the human flexor muscles during grasping, potentially complementing weak motor functions.

The assistive capability of the Soft Synergy-Based Robotic Glove was assessed in a grasping task study executed by a patient suffering from an incomplete C7 SCI. The subject (male, 58), demonstrated severe impairment in his dominant hand (right) as well as moderate motor impairment of his nondominant hand.

The assessment was conducted at the Spinal Unit of Careggi Hospital, Florence, Italy, in according to the ethical principles for medical research (WMA declaration of Helsinki), and the subject signed a written informed consent for participating in the study. The primary performance metric used in this case study experiment was the Box and Block manual dexterity test (Mathiowetz et al., 1985). The patient completed the tests and was observed under two conditions: with and without the exosuit—called “glove” and “no-glove” conditions, respectively.

**Figure 9.**
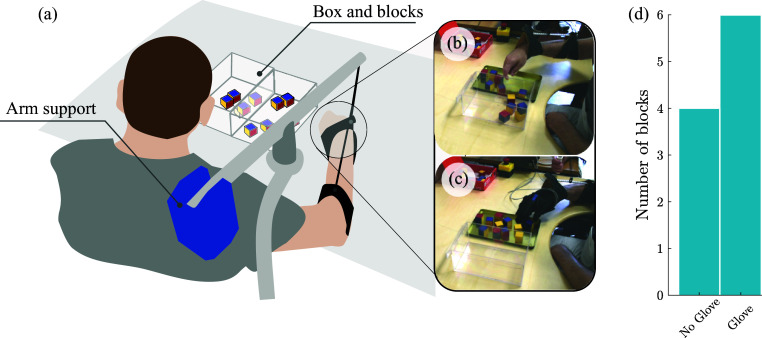
(a) Subject of single-use case study performing the Box and Block test. The counterweight sling attachment prevented arm fatigue throughout the experiment. (b) Photo of the subject in the “glove” condition. (c) Photo of the subject in the “no-glove” condition. (d) Summary of subject’s performance in both cases.

The patient sat in his own wheelchair which, for the purposes of this experiment, had been modified with an additional support structure. The counterweight support system, shown in Figure 9, helped mitigate the subject’s arm fatigue throughout the experiment.

The patient sat at a height-adjusted table, on top of which two boxes were presented to him. The leftmost box was empty, and the right-hand box was filled with square blocks. In accordance with the Box and Block test, the patient was instructed to transfer as many blocks as possible from the right box to the left one, in a fixed period of 60 s, using only his right arm. For each condition, the subject performed two trials, and before each trial, the patient waited at least 5-min and received a 1-min stretching massage in order to avoid fatigue and to start from a relaxed condition. To operate the glove, the subject communicated the desire to close or release the hand to the operator. The subject performed the trials without glove assistance before the trials examining glove assistance.

The results of the case study indicate that the use of this device has the potential to improve grasping performance of a motor-compromised wearer. In the control condition (no-glove), the patient was capable of transferring four blocks with no assistance. When utilizing glove assistance, the subject increased the number of blocks moved to six. This performance boost, taken on its own, is moderate. However, it is notable that throughout the initial 30 s of both cases (one half of the test time), the subject had moved the same number of blocks (three). In the latter 30 s of the test, the subject was only able to place one block without glove assistance. When using glove assistance during this portion of the test, he was able to place three additional blocks in this same period. These results are summarized in Table 2.

**Table 2.** Experimental results of subject performance during the Box and Block case study

	Initial 30 s		Final 30 s	
	# of blocks	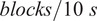	# of blocks	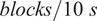
No glove	3	1	1	0.33
Glove	3	1	3	1

This improvement in performance throughout the latter portion of the test indicates a possible delay in fatigue onset of the patient. Notably, the rate of block transfer remained roughly the same throughout the entirety of the Box and Block test with assistance, whereas the rate of block transfer fell dramatically when the subject was provided no assistance.

## Discussion

The incredible dexterity and maneuverability of the human hand allows for the performance of a high number of activities with great precision. Unfortunately, too many people experience the drastic reduction in quality of life that comes with the loss of proper manual control. Many approaches to the restoration and rehabilitation of the hand after an injury attempt to utilize high degrees of control to replicate this lost dexterity directly. The method proposed in this work takes a different approach, reducing the number of control inputs required while simultaneously allowing for the existence of more complex assistance kinematics.

A growing body of literature suggests the existence of a subset of motion synergies, which are both shared across subjects and low in DoF, from which a majority of common actions may be reconstructed (Ingram et al., 2008; Santello et al., 1998). The identification and exploration of these common grasp synergies has continued to develop as an important topic of research in recent years (Burns and Vinjamuri, 2020; Gracia-Ibáñez et al., 2020). With wide-ranging applications and implications in the rehabilitation and assistive living fields, data on this topic have only continued to become more accessible (Jarque-Bou et al., 2020).

We have exploited this idea to implement a simple mechanism that assists closing of the human hand with a single electric motor. The main limitation of our approach is dexterity: by allowing only one pattern of closure of the hand, although the one that by definition allows for most of our daily tasks, we cannot individually control DoFs of the hand.

This hard constraint is softened by the compliance of the transmission, allowing for a larger range of hand postures to be realized. Nevertheless, a slight change in the way forces are distributed across fingers was observed between an assisted grasp and an unassisted grasp (Figure 7). A differential adaptive mechanism, similar to the one presented in (Catalano et al., 2014) for a prosthetic hand or in (In et al., 2015) for a soft robotic glove, in combination with the first postural synergy, might yield better results.

### Transmission Effectiveness

When adapting this synergy-based approach to a wearable device, it is important to consider precisely how accurately these grasp synergies are being delivered to the hand. In this regard, it is important to consider that despite having multiple advantages over a rigid system, exosuits inherently transmit compressive loads through the skeletal structure of the wearer, and as a result, the total amount of assistance that can be provided is limited. In addition to this, energy is lost during suit loading. In tendon-driven systems, this energy is dissipated at many stages of power transmission, although primarily through the stretching of the tendons, the compression of the sheath, the stretching of the textile, and the deformation of the user tissue (Asbeck et al., 2015). Although not much can be done regarding the deformation of the human system, if the design is to be improved upon, it is important to understand where within the exosuit system the energy losses are greatest and where losses within the human system can be minimized.

Because the tendon network on the glove itself is rather direct, it was hypothesized that the bulk of the energetic losses of this system would arise from the arrangement of the transmission elements between the actuator and the glove itself. This hypothesis is supported by transmission analysis of other exosuit devices, which found that the majority of negative work arose during the loading of interface components (Yandell et al., 2017). In this regard, Figure 5d describes the stress–strain relationship of both major transmission elements: the tendon unit and the Bowden coil.

Both elements that were tested demonstrated strong hysteretic characteristics throughout each loading cycle. Although it should be noted that, in the case of the tendon analysis, the slope of the stress–strain curve is approximately the same throughout loading and unloading. Therefore, although hysteresis was observed, it is the linear characteristics which dominate its stress–strain response. This is good for controllability; although hysteresis is present, it does not manifest within the tendon as a loss or change of stiffness. It is possible that a fraction of the hysteresis bands shown in Figure 5d materialized as an artifact of the system backlash (Figure 5c).

With this possibility in mind, these data show that the tendons used in this system are more aptly modeled as linear, high-stiffness springs, rather than inextensible, force transmission elements. The hysteresis bands observed here align with those seen in characterizations of other wearable devices (Quinlivan et al., 2015). It is expected that the precise shape of these hysteresis curves, and the amount of energy lost from this behavior, will vary subject to the organization of the cables and curvature of the loading paths on the device itself (Jeong and Cho, 2019). This implies that special care should be taken in design to minimize total bend angle and ensure that the amount of energy lost as a result can be minimized.

The system backlash, shown in Figure 5c, was characterized fairly accurately, with high repeatability and very low standard deviations over many cycles (*n* = 10). When backlash is understood to this degree, it is possible to compensate for this effect through control with high accuracy (Dinh et al., 2017b).

At the onset of loading, the there exists a “dead zone” of about 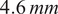 in which inputs to the tendon are not represented at the Bowden coil output. This happens as the tendon tightens itself around the curvature of the Bowden sheath, as illustrated in the subfigures of Figure 5c. A similar “dead zone” occurs throughout unloading of the tendon. This cyclical dead zone introduces serious problems in terms of maximum control bandwidth as well as a lower limit to the control inputs that the system will respond to. And, of course, this dead zone exists only as a result of the Bowden sheath geometry. In practical wear conditions, the degree of backlash will change with the geometry and bend angle of the transmission, as the user moves and changes position (Jeong and Cho, 2019). This must be adapted for through control. Methods for detecting these changes have been recently presented and will be useful in future development of wearable, tendon-driven devices (Jeong and Cho, 2016).

The insights gained from these tests have broad implications for the optimal design of load-bearing wearable devices. To reduce backlash, special care should be taken when routing Bowden cables of the tendon-drive system to minimize bending angles. Internal compliance within the tendon-driven transmission may be limited by use of thicker tendons, or tendons of different materials which have a higher yield tolerance. Stiffer sheaths composed of reinforced materials may also help in this regard. The characterization of these transmission elements would be helpful for the design of a controller to finely modulate grasping force (while holding an object) or hand position (in free-space or in the reaching phase; Dinh et al., 2017b).

It may also be worth exploring other means of system actuation to reduce the compliance imposed by the tendon-driven transmission. A flexible shaft-driven system, in which the tendon network exists solely on the glove, may substantially reduce these effects. It may be possible to further reduce energy lost to textile stretch by replacing the textiles of the glove with flexible, polymeric materials which fold comfortably around the skin, yet have high tensile strength.

### Pilot-Use Case Study

In spite of these inefficiencies, force production at the finger output remained predictable for both pinch and cylindrical grasp types (Figure 7d,e), even in high-current conditions. This near-linear relationship that was obtained between motor current and output force has promising implications for the controllability of the system.

However, equally important is that the actuation of the glove does not interfere with or alter the natural grasp dynamics of the wearer while interacting with the environment. Figure 7c shows that the distribution of grasp pressure observed in a healthy subject was modified by only marginal amounts when wearing the device. While a deeper analysis of this relationship is required, including the examination of different grasp types and object geometries, this finding supports the hypothesis that utilizing grasp synergies through design can provide necessary and complimentary user assistance while limiting disruption to the natural dynamics of the wearer.

Within the limited context explored here, the subject verbally reported good agreement with the device during use. The outcome results of this case study, although moderate, imply that through the use of the assistive device presented here, fatigue onset can be delayed in a motor-compromised user. This is best understood by examining the latter half of the Box and Block test (Table 2). Without the added assistance from the glove, the subject struggled to place only a single additional block during this period. Whereas after receiving assistance throughout the initial 30-s period, the subject was capable of transferring three additional blocks, maintaining the same rate of block transfer as in the initial 30-s period of both tests.

In addition, wearing of the glove induced significant changes in the subject’s grasping strategy. During the unassisted portion of the experiment, the patient utilized a grasping method best described as a blend between the lateral pinch and span grasp types (Feix et al., 2015) by gripping each block between the lateral surfaces of the thumb and index finger. While assisted, however, the patient shifted to that of a precision pinch grasp, in which the distal phalanxes of the thumb, index, and middle fingers were in contact with the block. It is believed that the presence of the passive dorsal springs made this possible by coercing his hand into a more apt posture when relaxed.

Furthermore, this pilot study gave important insights into the usability and comfort of the device. Donning of the glove was delicate due to residual spasticity of the patient’s hand and required about 5 min with assistance. Doffing of the glove, with clinician assistance, was simpler and took less than 1 min. After the experiment, although the subject expressed no discomfort, there were some mild and localized pressure marks on his hand due to the fit of the glove. For this reason, additional focus on further increasing wearability and comfort of the glove will be made in future iterations.

Of course, these results are preliminary. Further validation of the device effectiveness across a wider subset of patients is required. Whether due to differences in rehabilitation, or fundamental differences in initial injury, most patients suffering from SCI adopt differing strategies for executing ADLs; there is little guarantee that this device will prove as effective for other patients as for the subject of this case study. More in-depth clinical trials are undoubtedly required. Additionally, it will be interesting to study how a subject’s performance may evolve by using the glove over longer periods of time or throughout other, more demanding tests, such as the Nine-Hole Peg Test, for example (Grice et al., 2003).

### Wider Implications

Many systems designed for targeted rehabilitation and augmentation of the hand focus on applying agency to many DoFs in order to accurately replicate a wide variety of poses and grasps (Polygerinos et al., 2015; Rose and O’Malley, 2018; Gerez et al., 2020). These types of solutions have incredible potential to create assistive solutions with a degree of dexterity rivaling that of a healthy human hand. Yet, these additional DoFs add additional layers of complexity to design and control. This is all before considering the additional mass and bulk which must be added to the system itself to accommodate these DoFs.

One such solution, proposed by the MAHI Lab (Rose and O’Malley, 2018), boasts an impressive number of degrees of control. Yet, the number of actuators required to enforce these motions add considerable weight and power requirements. An additional consideration is the advanced control techniques which must be used to control such a complex system. A synergy-based approach, such as the solution proposed here, can still provide assistance for a large number of ADLs while significantly reducing the number of actuators required and enhancing user comfort through the recruitment of biomimetic motion patterns.

Other underactuated solutions have been proposed as well (Table 3). The HERO Glove (Yurkewich et al., 2019), from the Intelligent Assistive Technology and Systems Lab, drives all five digits from a single, nonbackdrivable motor. The distal mounting of this actuator, however, adds significant bulk and mass to the hand. The exo-glove developed by the New Dexterity research group (Gerez et al., 2019) allows each finger to converge to different closing positions through the use of a novel differential mechanism. While this adaptation may accurately bring the fingers to proper and comfortable positions during a grasp, this approach will not replicate the different retraction velocities of a true synergy-based method, such as is proposed here. In a similar vein, the underactuated approaches of the Exo-Glove Poly and Exo-Glove Poly II rectify the difference in the retraction forces of each finger through the intrinsic elasticity of their polymer construction (Kang et al., 2019).

**Table 3.** State-of-the-art comparison of underactuated, tendon-driven glove systems

Design characteristic	This design	Exo-glove poly II^1^	HERO glove^2^	SEM glove^3^
Supported fingers	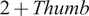	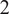	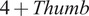	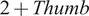
Fingertip force (N)	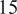	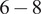	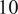	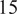
Distal mass (g)	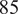	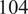	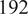	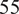
Construction	Fabric	Polymer	Fabric	Fabric
Finger extension	Passive	Active	Active	None

1 (Kang et al., 2019).

2 (Yurkewich et al., 2019).

3 (Gerez et al., 2019).

The solution proposed in this work presents a preliminary path forward toward achieving noteworthy levels of dexterity while maintaining low complexity through the incorporation of biomimetic postural synergies into the system design. Although underactuated, the robotic glove proposed here is capable of providing comfortable grasp assistance for a broad variety of common items (Figure 4a–g), allowing for the potential of an improved quality of life for the user. This design, based around the utilization of common postural synergies, demonstrates the practical viability of generating different joint velocities from a single actuator. It is the authors’ belief that an underactuated system which is designed to actuate two or even three of the most commonly recruited postural synergies would be capable of providing a remarkably high variety of assistance throughout common, everyday tasks.

## Conclusions and Future Directions

In this work, the design and characterization of a novel wearable device was presented along with the results of a single-use case study. The synergy-based robotic glove, designed around the principle of postural synergies, is low-profile, lightweight, and demonstrates that the incorporation of postural synergies offers the potential to increase device adaptability while simultaneously reducing complexity of control and power transmission. Furthermore, this implementation of postural synergy kinematics into the transmission of the device had little effect on the final distribution of grasp pressures observed at the fingertip.

In a use-case scenario, the glove was able to be donned and doffed by the case study patient with physician assistance within a 5-min period. Patient performance during the Box and Block test showed a marked 50% improvement when compared to the control condition. Significantly, it appears as if the use of the glove is related to a delay in fatigue onset, as observed in the latter half of the test.

There are numerous avenues which could be explored to develop this concept further in the near future. It is primary to investigate the benefits of an increased variety of supported postural synergies on the device, and importantly, how effective each supported synergy is in expanding the variety of everyday tasks the glove is capable of assisting. Another critical step forward is to explore more interesting solutions for control. A neurological-based control, for example, would allow for greater patient freedom during rehabilitation and make the device into a more useful rehabilitation tool. This would allow for the device to be potentially used by more severely compromised patients, while also attempting to take full advantage of the capabilities offered by multiple postural synergies acting in tandem.

## Data Availability

The datasets used and/or analyzed during the current study are available from the corresponding author upon reasonable request.
